# High Entrepreneurship, Leadership, and Professionalism (HELP): A New Resource for Workers in the 21st Century

**DOI:** 10.3389/fpsyg.2018.01480

**Published:** 2018-08-28

**Authors:** Letizia Palazzeschi, Ornella Bucci, Annamaria Di Fabio

**Affiliations:** Department of Education and Psychology, Psychology Section, University of Florence, Florence, Italy

**Keywords:** entrepreneurship, leadership, professionalism, personality traits, workplace relational civility, flourishing, entrepreneurs, workers

## Abstract

World of work in the 21st century is characterized by instability, insecurity, and continuous change. To face these challenges of the post-modern era, workers are required to use their personal resources. A new construct called high entrepreneurship, leadership, and professionalism (HELP) is a preventive resource that helps maintain, improve, and find work in uncertain or dynamic conditions. This study aims to examine the personality correlates of HELP in Italian workers and identify different clusters based on HELP and other variables, such as workplace relational civility and flourishing. To this end, the following instruments were administered to 204 Italian workers: the HELP questionnaire, the Big Five Questionnaire, the Workplace Relational Civility Scale, and the Flourishing Scale. The personality correlates of HELP underscored the role of conscientiousness (and its subdimension perseverance) and extraversion (and its subdimension dominance). The cluster analysis identified three clusters characterized by high, average, and low HELP scores. Participants in the first cluster with high HELP scores appeared to possess higher perseverance, dominance, workplace relational civility, especially readiness, and higher flourishing than those in the other two groups. The present results can open new opportunities for future research and interventions in a primary prevention perspective to foster resources for workers and healthy organizations in the 21st century.

## Introduction

World of work in the 21st century is characterized by instability, insecurity, and continuous change, and navigating this complex work scenario requires workers to develop their personal resources (Buunk et al., 2007; Peiró et al., 2010; Silla et al., 2010; Savickas, 2011; Guichard, 2013; Di Fabio and Kenny, 2016a,b; De la Fuente et al., 2017a,b). A new construct, high entrepreneurship, leadership and professionalism (HELP; Di Fabio et al., 2016) is considered a promising resource to actively construct career paths and help workers negotiate the challenges of the present liquid society (Bauman, 2000). Entrepreneurship, leadership, and professionalism have emerged as core constructs, crucial in the 21st century (Di Fabio et al., 2016). Although they have been studied separately in the past, only recently have they been examined as an integrated construct (Di Fabio et al., 2016) that reflects aspects of motivation, intention, and efficacy. Di Fabio et al. (2016) have developed a theoretically and empirically integrated framework for studying HELP.

Entrepreneurship has been recently defined as “a process that evolves over time and includes different phases from forming an intention, starting-up, scaling-up, stabilizing, and managing the business, exit and potential re-entry” (Gorgievski and Stephan, 2016, p. 440). It is a highly valued concept in the 21st century because it can help create job opportunities and boost productivity and economic growth (Van Praag and Versloot, 2008). There are two approaches to defining entrepreneurship in the psychological literature (Gorgievski and Stephan, 2016): the first considers entrepreneurs as an occupational category that includes individuals who are self-employed and manage their own business; the second regards entrepreneurial action and processes as those that are implied in the individuation, construction, and implementation of opportunities (Shane and Venkataraman, 2000; Davidsson, 2015, 2016). The most recent psychological perspective on entrepreneurship (Gorgievski and Stephan, 2016) identifies three dimensions. The first dimension refers to within-individual processes, between-individual differences, and interactions between individual entrepreneurs and their immediate (teams, units, and organizations) and wider contexts (regions or countries). The second dimension refers to multi-level processes across different phases of the entrepreneurial process, and the third dimension focuses on different kinds of businesses in terms of the entrepreneur’s identity, goals, and start-up motivations.

Leadership is related to the broader theme of human resource management (Hitt and Duane, 2002; Peiró and Rodríguez, 2008; Monzani et al., 2015). It regards the influence of group activities to achieve an objective (Rauch and Behling, 1984; House et al., 1999; Boyatzis, 2008; Boyatzis et al., 2015). Leadership has traditionally been considered a process of influence between the leader and group members toward achieving group aims (Hollander, 1992). Two well-known leadership styles are transactional and transformational (Burns, 1978; Bass, 1985). Transactional leadership is characterized by the leader demonstrating initiative to connect with the group members in their work context for the exchange of resources. On the other hand, transformational leadership involves changes in the beliefs, needs, and values of the collaborators.

Most recently, new leadership styles have been proposed. Sustainable leadership aims to avoid social and environmental damage (Hargreaves and Fink, 2003, 2004); servant leadership focuses on the personal growth of the subordinates (Ehrhart, 2004); benevolent leadership involves leaders treating followers as family members, showing concern for their well-being in both the work domain and private life (Cheng et al., 2004; Wang and Cheng, 2010); authentic leadership uses a transparent and ethical leadership style, emphasizing people’s strengths rather than their weaknesses (Avolio et al., 2009); ethical leadership entails the pursuit of the right aims and focuses on empowering the organization’s members (Gallagher and Tschudin, 2010); and mindful leadership involves paying attention to the present moment, recognizing feelings and emotions, and keeping them under control, especially under stress (George, 2012). Entrepreneurial leadership is the basis of HELP construct. It considers the ability to influence others for using resources in a strategic manner with the aim of promoting behaviors that seek opportunities and advantages (Ireland et al., 2003), including setting clear goals, creating opportunities, empowering people, and promoting mutual and organizational awareness (Cunningham and Lischeron, 1991).

Professionalism is the third aspect of HELP, after entrepreneurship and leadership (Di Fabio et al., 2016). It is traditionally defined as “an ongoing process through which an individual derives a cohesive sense of professional identity by integrating the broad-based knowledge, skills, and attitudes within psychology with one’s values and interests” (Ducheny et al., 1997, p. 29). Professionalism is particularly relevant to entrepreneurial leadership, which refers to the ability to envision strategic scenarios that can facilitate the identification and implementation of value creation (Gupta et al., 2004).

In constructing this framework, Kanter’s (1989) theory focused on the link between careers and economic, social, and political issues. Chan et al.’s (2012) framework adopted a person-centered perspective that highlighted subjective careers (Di Fabio et al., 2016). According to Kanter (1989) and Chan et al.’s (2012), in an unstable work environment, it is possible to recognize entrepreneurship, leadership, and professionalism (ELP) as three fundamental dimensions of a subjective career space. While Kanter (1989) maintained that these constructs are distinct, Chan et al. (2012) examined the motivation, intentions, and efficacy of each of these three different constructs. Subsequently, Di Fabio et al. (2016) proposed an integrated model where entrepreneurship, leadership, and professionalism were integrated into the HELP construct, along with the three aspects of motivation, intentions, and efficacy for each aspect. This integrated construct, HELP, seems particularly promising not only because it introduces a new preventive integrated perspective, but also because it is an increasable resource differently from personality traits that are considered substantially stable in the literature (Palazzeschi et al., 2018). From a primary preventive perspective (Hage et al., 2007; Kenny and Hage, 2009; Di Fabio and Kenny, 2016a), HELP can be seen as a resource for workers to manage the complex career challenges of the 21st century (Di Fabio et al., 2016). HELP can also promote healthy organizations (Lowe, 2010; Tetrick and Peiró, 2012; Di Fabio, 2016, 2017a; Di Fabio et al., 2016) where individuals have the resources to cope with the continuously changing work sphere through proactive and innovative solutions, which result in greater well-being.

### Aims and Hypotheses

The present study attempts to examine the personality correlates of HELP in Italian workers and identify different clusters based on HELP and other variables, such as workplace relational civility and flourishing, that are interesting variables in the framework of positive healthy organizations.

The three variables of entrepreneurship, leadership, and professionalism in relation to Big Five personality traits are examined in the literature considering them as separate constructs. We examined recent studies and reviews of the relationships between entrepreneurship and the Big Five personality traits. One meta-analysis that considered studies conducted between 1990 and 2010 pointed to the presence of positive relationships between entrepreneurs’ performance and Conscientiousness, Openness, Extraversion, and a negative relationship between entrepreneurs’ performance and Neuroticism (Brandstätter, 2011). In Slovenian context, Antoncic et al. (2015) showed that entrepreneurship in terms of firm start-up activities is strongly and positively associated with Openness, less so with Extraversion, and inversely associated with Agreeableness.

Bono and Judge’s (2004) meta-analysis examined the relationships between the Big Five personality traits and transformational leadership. They examined 384 correlations from 26 independent studies and found the strongest positive correlation between transformational leadership and Extraversion, and a negative correlation between transformational leadership and Neuroticism. Authentic leadership was positively correlated with Conscientiousness and Openness to experience (Komariah, 2016). Furthermore, ethical leadership was inversely related to Neuroticism and positively to Openness to experience, Agreeableness, and Conscientiousness. Notably, ethical leadership was not correlated with Extraversion (Özbağ, 2016).

Extant research on the relationship between professionalism and personality traits is limited. One study showed a positive correlation between the professionalism of physician-assistant students and Conscientiousness (Moser and Dereczyk, 2012). A more recent study on anesthetist trainees also highlighted the association between professionalism and Conscientiousness (Sawdon et al., 2017).

In summary, previous research has regularly found a relationship between Extraversion, one of the Big Five traits and entrepreneurship, while Conscientiousness has been linked to leadership styles and professionalism. The other three Big Five personality traits—Agreeableness, Neuroticism, and Openness—were not conclusively linked with any of the three HELP constructs. Further, in a recent Italian study (Palazzeschi et al., 2018), involving workers of different public and private organizations, Conscientiousness emerged as the best personality correlate of HELP, followed by Extraversion.

Workplace relational civility (WRC, Di Fabio and Gori, 2016a) is a new kind of relational style in the workplace “characterized by respect and concern for oneself and others, interpersonal sensitivity, personal education, and kindness toward others. It also includes civil behaviors such as treating others with dignity and respecting social norms to facilitate peaceful and productive cohabitation” (Di Fabio and Gori, 2016a, p. 2). The WRC construct comprises three dimensions: (1) relational decency at work, which refers to decency-based relationships characterized by respect for oneself and others, assertiveness, ability to express beliefs and opinion, and relational capacity; (2) relational culture at work, which refers to politeness, kindness, good education, courteousness; and (3) relational readiness at work, which refers to sensibility toward others (speed in understanding the feelings of others and exhibiting proactive sensibility), ability to understand the emotions of others, concerns for others, delicacy, attention to the responses of others, empathy, and compassion. It is important to emphasize that WRC can be evaluated using a “mirror” scale of measurement—the Workplace Relational Civility Scale (Di Fabio and Gori, 2016a). Participants are first asked to indicate their relationship with others within a given period and then evaluate others’ relationships with them. This modality helps them recognize their self-importance in the process. WRC is an important positive variable that facilitates early intervention at the workplace. It is especially significant and innovative as previous studies have only examined the negative aspects of workplace incivility (Cortina et al., 2001).

Flourishing is an important positive variable that is a comprehensive measure of well-being, vital to workers in the 21st century. It is a form of eudaimonic well-being, perceived as success in relationships, purpose, and future optimism (Diener et al., 2010).

This study attempted to replicate an earlier study that examined the personality correlates of HELP among Italian workers in care organizations. On the basis of the previously described framework, the following hypotheses were formulated for this study.

Ha1: Among the Big Five personality traits, Conscientiousness will be most highly and positively correlated to HELP.
Ha2: Positive correlations will emerge between HELP and Extraversion.
Ha3: Agreeableness, Neuroticism, and Openness will not significantly correlate with HELP.

The second aim of the present study was to investigate the relationship of HELP also with workplace relational civility and flourishing, examining whether participants clustered in meaningful ways based upon HELP scores and personality traits, workplace relational civility, flourishing. Given the exploratory nature of this investigation, no specific hypotheses were advanced.

## Materials and Methods

### Participants

The study participants comprised 204 Italian workers employed in care organizations in the Tuscany region (female = 63.71%, male = 36.29%; mean age = 40.36 years, *SD* = 13.00).

### Measures

#### High Entrepreneurship, Leadership, Professionalism Questionnaire (HELP-Q)

The HELP-Q (Di Fabio et al., 2016) is a 9-item integrated scale that identifies entrepreneurship (E), leadership (L), and professionalism (P) in terms of motivations, intentions, and efficacy. The nine items—three for each area—are scored using a 5-point Likert-type scale (1 = *not at all*, 2 = a little, 3 = *somewhat*, 4 = *much*, 5 = *a great deal*). Examples of items include “To what extent is it important for me to look for new ideas on how to make a profit for entrepreneurship?” (Entrepreneurship). “To what extent is it important for me to become a leader or a manager?” (Leadership). “To what extent is it important for me to excel in my chosen area of study/work?” (Professionalism). Cronbach’s alpha coefficients were 0.92 for entrepreneurship, 0.92 for leadership, 0.90 for professionalism, and 0.77 for the total score.

#### Big Five Questionnaire (BFQ)

The BFQ (Caprara et al., 1993) comprises 132 items scored on a 5-point Likert scale, ranging from 1 = *Absolutely false* to 5 = *Absolutely true*. Cronbach’s alpha coefficients for the five personality traits were: 0.81 for Extraversion (e.g., “I think that I am an active and vigorous person”); 0.73 for Agreeableness (e.g., “I understand when people need my help”); 0.81 for Conscientiousness (e.g., “I tend to be very thoughtful”); 0.90 for Emotional stability (e.g., “I do not often feel tense”); 0.75 for Openness (e.g., “I am always informed about what is happening in the world”).

#### Workplace Relational Civility Scale (WRCS)

The WRCS is a 26-item self-report mirror instrument (Di Fabio and Gori, 2016a), covering three dimensions: relational readiness (RR), relational culture (RCu), and relational decency (RD) at work. The sum of these dimensions gives an overall score of WRC as well as a score for part A and B of the WRCS. Part A is the analysis of an individual’s self-perception as pertaining to a particular issue (e.g., “I was able to express my values and beliefs calmly to others”), and part B is the analysis of an individual’s perception of others on the same issue (e.g., “Others were able to express their values and beliefs calmly to me”). The participants were asked to describe their general relationship with others during 3 months prior to the administration and then describe their perception of others’ general relationship with them over the same period. Items were scored on a 5-point Likert scale (1 = *not at all*, 2 = *a little*, 3 = *somewhat*, 4 = *much*, 5 = *a great deal*). Cronbach’s alpha coefficients for the three dimensions within Part A were as follows: Factor RR (α = 0.83), Factor RCu (α = 0.76), and Factor RD (α = 0.75). Cronbach’s alphas for Part B were as follows: Factor RR (α = 0.86), Factor RCu (α = 0.88) and Factor RD (α = 0.85). The Cronbach’s alpha coefficients for the total scores for Part A and Part B were α = 0.87 and α = 0.92, respectively.

#### Flourishing Scale (FS)

The Italian version (Di Fabio, 2016) of the FS (Diener et al., 2010) was used to evaluate flourishing as a measure of eudaimonic well-being. The scale comprises eight items scored on a 7-point Likert scale ranging from 1 (*strongly disagree*) to 7 (*strongly agree*). Some examples of the items are as follows, “My social relationships are supportive and rewarding,” “I lead a purposeful and meaningful life,” “I am optimistic about my future.” The FS has a unidimensional structure and good reliability (α = 0.88).

### Procedure and Data Analysis

Questionnaires were administered by trained psychologists to participants in groups. The order of administering was counterbalanced to control possible effects of a fixed order of presenting the questionnaires. The study assured to respondents anonymity and confidentiality. The questionnaire included a statement regarding the personal data treatment, in accordance with the Italian privacy law (Law Decree DL-196/2003). The workers authorized and approved the use of anonymous/collective data for possible future scientific publications. Because the data was collected anonymously and the research investigated psycho-social variables not adopting a medical perspective, ethical approval was not sought.

Descriptive statistics and Pearson’s correlations were calculated. A cluster analysis (k-mean method) was also performed, and ANOVAs using Bonferroni *post hoc* tests were conducted.

Cluster analysis was carried out to individuate different groups on the basis of the different aspects of HELP and, therefore, to differentiate the groups with respect to the variables considered: personality traits, workplace relational civility, and flourishing. Specifically, we are interested in differentiating groups on the basis of combinations of scores on the three aspects of HELP and to analyze how they are different with respect to the studied variables. Cluster analysis identifies groups or types of individuals who share particular attributes or relations among attributes according to a person-center approach to scientific research (Bergman et al., 2003; Magnusson, 2003).

## Results

The results identified two personality correlates of HELP: Conscientiousness, (and its subdimension Perseverance) and Extraversion, (and its subdimension Dominance) (see **Tables 1**, **2**).

**Table 1:** Correlations between HELP and Big Five Dimensions (*N* = 204).

	1	2	3	4	5	6
1. HELP total	_					
2. BFQ extraversion	0.31^∗∗^	_				
3. BFQ agreeableness	0.01	0.22^∗∗^	_			
4. BFQ conscientiousness	0.37^∗∗^	0.50^∗∗^	0.19^∗∗^	_		
5. BFQ emotional stability	0.02	0.16^∗^	0.28^∗∗^	0.10	_	
6. BFQ openness	0.16	0.44^∗∗^	0.43^∗∗^	0.45^∗∗^	0.40^∗∗^	_

*HELP = High Entrepreneurship, Leadership, and Professionalism; BFQ = Big Five Questionnaire. ^∗^p < 0.05; ^∗∗^p < 0.01.*

**Table 2:** Correlations between HELP and Big Five Sub-Dimension Scores (*N* = 204).

	1	2	3	4	5	6	7	8	9	10	11
1. HELP total	_										
2. BFQ dynamism	0.27^∗∗^	_									
3. BFQ dominance	0.33^∗∗^	0.39^∗∗^	_								
4. BFQ cooperativeness	0.07	0.43^∗∗^	-0.02	_							
5. BFQ cordiality	-0.05	0.24^∗∗^	-0.09	0.53^∗∗^	_						
6. BFQ scrupulosity	0.20^∗∗^	0.28^∗∗^	0.22^∗∗^	0.16^∗^	0.08	_					
7. BFQ perseverance	0.35^∗∗^	0.55^∗∗^	0.34^∗∗^	0.26^∗∗^	0.09	0.50^∗∗^	_				
8. BFQ emotions control	0.03	0.23^∗∗^	0.12	0.13	0.08	0.02	0.23^∗∗^	_			
9. BFQ impulse control	0.07	0.05	0.05	0.32^∗∗^	0.34^∗∗^	0.05	-0.04	0.46^∗∗^	_		
10. BFQ openness to culture	0.09	0.41^∗∗^	0.11	0.37^∗∗^	0.31^∗∗^	0.29^∗∗^	0.26^∗∗^	0.24^∗∗^	0.37^∗∗^	_	
11. BFQ openness to experience	0.19^∗∗^	0.50^∗∗^	0.19^∗∗^	0.33^∗∗^	0.30^∗∗^	0.31^∗∗^	0.51^∗∗^	0.27^∗∗^	0.29^∗∗^	0.52^∗∗^	_

*HELP = High Entrepreneurship, Leadership, and Professionalism; BFQ = Big Five Questionnaire. ^∗^p < 0.05; ^∗∗^p < 0.01.*

Correlations among the dimensions of HELP are reported in **Table 3**.

**Table 3:** Correlations among the dimensions of HELP.

	1	2	3
1. HELP entrepreneurship			
2. HELP leadership	0.77^∗∗^		
3. HELP professionalism	0.67^∗∗^	0.73^∗∗^	

*HELP = High Entrepreneurship, Leadership, and Professionalism; ^∗∗^p < 0.01.*

Three groups were identified from the cluster analysis of HELP scores. Participants in Cluster 1, with high HELP scores, also had higher mean scores on each of the HELP aspect—entrepreneurship, leadership, and professionalism—than participants in Clusters 2 and 3, with average and low HELP scores, respectively (see **Table 4**).

**Table 4:** Three Emergent Clusters according to Overall HELP Scores and its Dimensions.

	Cluster 1 (*n* = 56)		Cluster 2 (*n* = 77)		Cluster 3 (*n* = 71)		*F*(2,201)
	*M*	*SD*	*M*	*SD*	*M*	*SD*	
HELP total	38.68bc	3.30	28.90ac	3.21	18.14ab	3.52	594.75^∗∗∗^
HELP entrepreneurship	12.55bc	1.93	9.21ac	1.76	5.20ab	1.49	551.37^∗∗∗^
HELP leadership	13.14bc	1.24	9.23ac	1.20	5.06ab	1.61	289.59^∗∗∗^
HELP professionalism	12.92bc	1.57	10.47ac	1.69	7.90ab	2.02	406.62^∗∗∗^

*HELP = High Entrepreneurship, Leadership, and Professionalism. a = Cluster 1, b = Cluster 2, c = Cluster 3, ^∗∗∗^p < 0.001. Bonferroni post hoc tests.*

ANOVAs relative to personality traits, WRC, and flourishing showed significant differences in terms of external descriptor variables (see **Table 5**).

**Table 5:** ANOVAs for Cluster 1, Cluster 2, and Cluster 3 relative to HELP Scores, Personality Traits, WRC, and Flourishing.

	Cluster 1 (*n* = 56)		Cluster 2 (*n* = 77)		Cluster 3 (*n* = 71)		*F*(2,201)
	*M*	*SD*	*M*	*SD*	*M*	*SD*	
BFQ extraversion	77.23bc	7.31	74.12ac	7.84	68.78ab	7.25	21.21^∗∗∗^
BFQ agreeableness	78.13	8.24	77.77	8.15	77.39	8.10	.13
BFQ conscientiousness	83.96bc	8.65	79.71ac	11.04	76.83ab	9.63	8.08^∗∗∗^
BFQ emotional stability	68.03	11.35	70.13	9.86	67.90	11.65	0.95
BFQ openness	79.23	10.78	79.61	10.92	76.21	9.30	2.59
BFQ dynamism	40.20c	5.01	39.23c	5.63	36.20ab	5.10	10.35^∗∗∗^
BFQ dominance	37.07	4.04	34.88	3.87	32.58	3.82	20.94^∗∗∗^
BFQ cooperativity	41.54	4.51	40.73	4.25	40.44	4.08	1.09
BFQ cordiality	36.59	4.84	37.04	5.05	36.97	5.02	0.14
BFQ scrupulosity	39.14	5.11	38.27	5.78	37.01	6.10	2.24
BFQ perseverance	44.82bc	5.43	41.44ac	6.57	39.82ab	5.26	11.76^∗∗∗^
BFQ emotional control	34.75	7.02	35.99	7.59	33.96	7.59	1.67
BFQ impulse control	33.29	5.97	34.14	5.74	33.94	6.22	0.35
BFQ openness to culture	38.29	6.80	39.66	5.14	37.83	5.38	2.05
BFQ openness to experience	40.95	5.84	39.94	5.15	38.38	5.42	3.63
WRC part A	54.18bc	6.05	49.55ac	8.31	45.86ab	7.56	19.32^∗∗∗^
WRC readiness part A	20.57bc	2.89	18.88ac	3.28	17.35ab	3.48	15.35^∗∗∗^
WRC culture part A	16.94bc	1.97	15.54a	2.88	14.87a	2.62	10.57^∗∗∗^
WRC decency part A	16.66bc	2.36	14.93ac	3.31	13.63ab	2.60	17.87^∗∗∗^
WRC part B	49.60bc	7.66	41.51	9.63	39.31ab	10.02	20.83^∗∗∗^
WRC readiness part B	18.29bc	3.66	15.26ac	4.72	14.69ab	3.95	12.97^∗∗∗^
WRC culture part B	16.27bc	2.32	12.86ac	3.36	12.75ab	3.35	25.23^∗∗∗^
WRC decency part B	15.05bc	2.67	12.87ac	3.08	11.87ab	3.48	16.63^∗∗∗^
Flourishing	46.64bc	5.81	40.44ac	6.64	37.15ab	7.14	32.73^∗∗∗^

*HELP = High Entrepreneurship, Leadership, and Professionalism; BFQ = Big Five Questionnaire, WRC = Workplace Relational Civility. a = Cluster 1, b = Cluster 2, c = Cluster 3, ^∗∗∗^p < 0.001. Bonferroni post hoc tests.*

Cluster 1 participants appeared to have greater Conscientiousness and Extraversion, with particularly high scores in the Perseverance and Dominance subdimensions. Further, they had a higher perception of WRC, especially readiness, and higher flourishing scores than the two other clusters. Cluster 2 participants had intermediate scores—in a range between those of participants from Clusters 1 and 3—in all dimensions. Finally, Cluster 3 participants scored the lowest on all dimensions.

## Discussion

This study attempted to examine the personality correlates of HELP among Italian workers and identify different clusters of individuals on the basis of HELP and other variables important for fostering positive healthy organizations such as WRC, and flourishing. Regarding the first aim relative to the relationship between HELP and personality traits, the present study showed that individuals with higher HELP scores were more conscientious and also extraverts, confirming also on Italian workers in care organizations the results of Palazzeschi et al. (2018) study. Conscientiousness is a personality trait that has traditionally been associated with two aspects of the integrated HELP construct: leadership and professionalism. On the other hand, extraversion has been associated with entrepreneurship, which lends support to the significant results for conscientiousness and extraversion in this study. Further, with regard to the Big Five subdimensions, we found that individuals with higher HELP scores were more perseverant and dominant, which is consistent with previous findings (Palazzeschi et al., 2018). No significant correlations were observed for Agreeableness, Neuroticism, and Openness, which supports our third hypothesis and is consistent with the previous study in the Italian context (Palazzeschi et al., 2018). Thus, the HELP construct appears to call on the personality traits of Conscientiousness—in terms of Perseverance—in pursuing one’s goals and Extraversion—in terms of dominance—which reflects the ability to influence, guide, and manage other people (Caprara et al., 1993; Di Fabio et al., 2016). These personality characteristics seem to be important for constructing the career paths of workers in the 21st century.

Regarding the second aim of the present study to investigate the relationship between HELP, and also WRC, and flourishing, the analysis identified the presence of three clusters. Cluster 1 participants had greater Conscientiousness and Extraversion (in terms of Perseverance and Dominance, respectively), higher WRC, particularly reflected in readiness (sensibility, ability to understand the emotions of others, concern for others, delicacy, attention to the responses of others, empathy, and compassion; Di Fabio et al., 2016), and higher flourishing in terms of perceived success in relationships, purpose, and future optimism (Diener et al., 2010). These results suggest that people with higher HELP levels are more conscientious (perseverant) and extroverted (dominant). They also pay more attention to others, which is expressed as a readiness to listen to others, and experience a form of well-being related to perceived success in relationships, a sense of purpose, and optimism. Thus, HELP represents a promising preventive resource in the 21st century and in its unpredictable world of work (Di Fabio and Palazzeschi, 2016) both for workers and healthy organizations (Lowe, 2010; Tetrick and Peiró, 2012; Di Fabio, 2016, 2017a,b; Di Fabio et al., 2016). It helps entrepreneurs and workers to proactively and innovatively manage these post-modern challenges (Di Fabio et al., 2017) while also considering aspects of WRC and flourishing.

Although this study examines the integrated construct of HELP and its personality correlates in depth and identifies different clusters of individuals on the basis of HELP scores, it is necessary to highlight some limitations of this study, particularly in relation to the characteristics of the participants. The participants were not representative of the national population because all the participants were from the Tuscany region of Italy. Future research could extend this study by including participants from different parts of Italy and from different organizations. This study can also be replicated in other international contexts. Future research could also continue to study in depth the relationships between the new integrated construct of HELP and personality traits to see if these results will be confirmed or will emerge specificities in relation to different categories and contexts. Notwithstanding the highlighted limitations, the results of this work offers new opportunities for future research and interventions from a primary prevention perspective (Hage et al., 2007; Kenny and Hage, 2009; Di Fabio and Kenny, 2016b; Di Fabio, 2017a) for entrepreneurs, workers, and healthy organizations in the 21st century (Tetrick and Peiró, 2012; Di Fabio and Palazzeschi, 2012; Di Fabio et al., 2016; Di Fabio and Kenny, 2016a; Di Fabio and Gori, 2016b; Dempsey and Kauffman, 2017; Di Fabio and Peiró, 2018). HELP can be considered as preventive individual resources to successfully face with the challenges of the current continuous changing labor market. The HELP highlights the value of personal entrepreneurship, leadership and professionalism as an integrated preventive core of characteristics that can adaptively build one’s one personal and professional paths. The HELP can also prevent possible career decision-making problems or failures rather than concentrating just on remediation. Furthermore the HELP calls for early actions to enhance personal resources in a primary prevention perspective (Hage et al., 2007; Kenny and Hage, 2009; Di Fabio and Palazzeschi, 2015; Di Fabio and Kenny, 2016b) to help individuals prevent future career and life problems.

The integrated construct of HELP appears to be promising resource as it can be developed and it facilitates early intervention, unlike personality traits that are considered to be stable. HELP represents a new integrated resource in a preventive perspective because the aspects it covers can be cultivated through early training. A key advantage of this resource is that the measurement scale is brief and easy to administer. The unstable and unpredictable world of work calls for new preventive resources to cope with multiple, differing challenges. In this framework, HELP can be considered an opportunity to find, maintain, and improve work in the 21st century (Bauman, 2000; Di Fabio et al., 2016).

## Author Contributions

LP and ADF conceptualized the study, chose the theoretical framework and the measures, analyzed the data, and wrote the methods and results. OB helped in the collection of the data. All authors wrote the paper together and read and revised the manuscript several times.

## Conflict of Interest Statement

The authors declare that the research was conducted in the absence of any commercial or financial relationships that could be construed as a potential conflict of interest.
